# Deep Infiltrating Endometriosis Malignant Invasion of Cervical Wall and Rectal Wall With Lynch Syndrome： A Rare Case Report and Review of Literature

**DOI:** 10.3389/fonc.2022.832228

**Published:** 2022-03-23

**Authors:** Baoxuan Li, Yang Wang, Yue Wang, Siman Li, Kuiran Liu

**Affiliations:** Department of Obstetrics and Gynecology, Shengjing Hospital of China Medical University, Shenyang, China

**Keywords:** endometriosis, deep infiltrating endometriosis, malignant transformation, Lynch syndrome, malignant tumor

## Abstract

**Background:**

Malignant transformation of deep infiltrating endometriosis (DIE) invading the cervix and rectum is quite rare, especially in patients combined with Lynch syndrome (LS). We report a rare case of a 49-year-old perimenopausal woman with endometrioid carcinoma arising from the pouch of Douglas, invading the cervix and rectum 1 year after a unilateral salpingo-oophorectomy treatment for ovarian endometriosis. The genetic testing of the patient showed germline mutations in MSH2, which combined with the special family history of colorectal cancer of the patient, was also thought to be associated with LS. We have analyzed the reported cases of DIE malignant transformation over the last 10 years, and reviewed the relevant literature, in order to strengthen the clinical management of patients with endometriosis, particularly patients with DIE, and reveal a possible correlation between malignant transformation of endometriosis and LS.

**Case Presentation:**

A 49-year-old perimenopausal woman presented with hypogastralgia, diarrhea, and intermittent fever for more than 1 month. A Transvaginal ultrasound (TVS) showed a cervix isthmus mass, and a magnetic resonance imaging (MRI) showed a mass in pouch of Douglas with high suspicion of malignancy, possibly invading the anterior wall of the rectum. Prior to surgery, the patient performed the ultrasound guided pelvic mass biopsy through the vagina, and the pathology of the mass showed endometrioid carcinoma. The patient received a gynecological–surgical laparotomy and enterostomy, and a histopathology revealed endometrioid carcinoma infiltrating the cervical wall and rectal wall. In the family genetic history of the patient, her mother and two sisters suffered from colorectal cancer, so lesion tissue and blood were taken for genetic testing, which showed a germline mutation in MSH2, with LS being considered. After the surgical treatment, the patient received six courses of paclitaxel–carboplatin chemotherapy. During the course of treatment, bone marrow suppression occurred, but was healed after symptomatic treatment. To date, the patient is generally in good health, and imaging examination showed no evidence of recurrence.

**Conclusion:**

The risk of malignant transformation of endometriosis is increased in perimenopause and postmenopause, as DIE is a rare malignant transformation of endometriosis. DIE can invade other adjacent organs and cause poor prognosis, thus, comprehensive gynecological–surgical treatment should be necessary. In addition, if histopathology showed endometrioid carcinoma, the possibility of LS should be considered, and if necessary, immunohistochemical staining and gene detection should be improved to provide follow-up targeted therapy and immunotherapy.

## Introduction

Endometriosis is a common benign disease in women of childbearing age, but it has malignant tumor-like behaviors such as invasion, implantation, and recurrence. Malignant transformation of endometriosis is rare, but the incidence rate is increasing (1, 2). There are different pathological types of endometriosis-related malignant tumors, and the common pathological types are endometrioid adenocarcinoma and clear cell carcinoma (3). The mechanism of endometriosis-associated malignant tumor is unclear, and current studies suggest an association with inflammatory response, hormone imbalance, oxidative stress, mutations in ARID1A, PIK3CA, KRAS or PTEN genes, loss of mismatch repair enzyme expression, and microsatellite instability. DIE is a rare type of endometriosis whose occurrence is considered to be related to somatic mutation. Malignant transformation of endometriosis mostly occurs in the ovarian endometriosis cyst, but rarely in DIE (4). The prognosis for endometriosis-associated malignant tumor is relatively better, but the prognosis for clear cell carcinoma usually is poor (1). Comprehensive surgical treatment and follow-up chemotherapy are mainly clinical treatment (5). The case we reported also considered the possibility of an LS, and the current literature shows that studies have been conducted to explore the relationship between LS, endometrioid adenocarcinoma, and clear cell carcinoma, but there is no systematic evaluation (1, 6, 7). There may be common molecular mechanisms between LS and endometriosis-associated malignant tumors. If necessary, immunohistochemical staining and gene detection can be added to endometriosis-associated malignant tumors to provide subsequent targeted therapy and immunotherapy. In the future, efforts should be made to develop effective and non-invasive screening tools to early identify women at risk of developing cancers (8).

## Case Report

A 49-year-old female, was hospitalized in the Gynecology Department of Shengjing Hospital Affiliated to China Medical University in February 2021, due to hypogastralgia, diarrhea, and intermittent fever for more than 1 month. She had undergone “right salpingo-oophorectomy, myomectomy and diagnostic curettage” for “right ovarian endometriosis cyst and uterine fibroid” one year prior to onset, and the pathology showed a “(right) ovarian endometriosis cyst and uterine fibroid”; menstruation resumed after 5 courses of GnRH-α treatment. The patient developed intermittent fever on January 2021, accompanied by hypogastralgia, was weak and also had diarrhea. She received anti-inflammatory treatment in the local hospital, but still had intermittent fever. The patient was transferred to our hospital for further treatment, where there was a weight loss of 2 kg during the period. Special family history: the mother and one sister of the patient had colon cancer, and another sister had rectal cancer.

**Figure 1:**
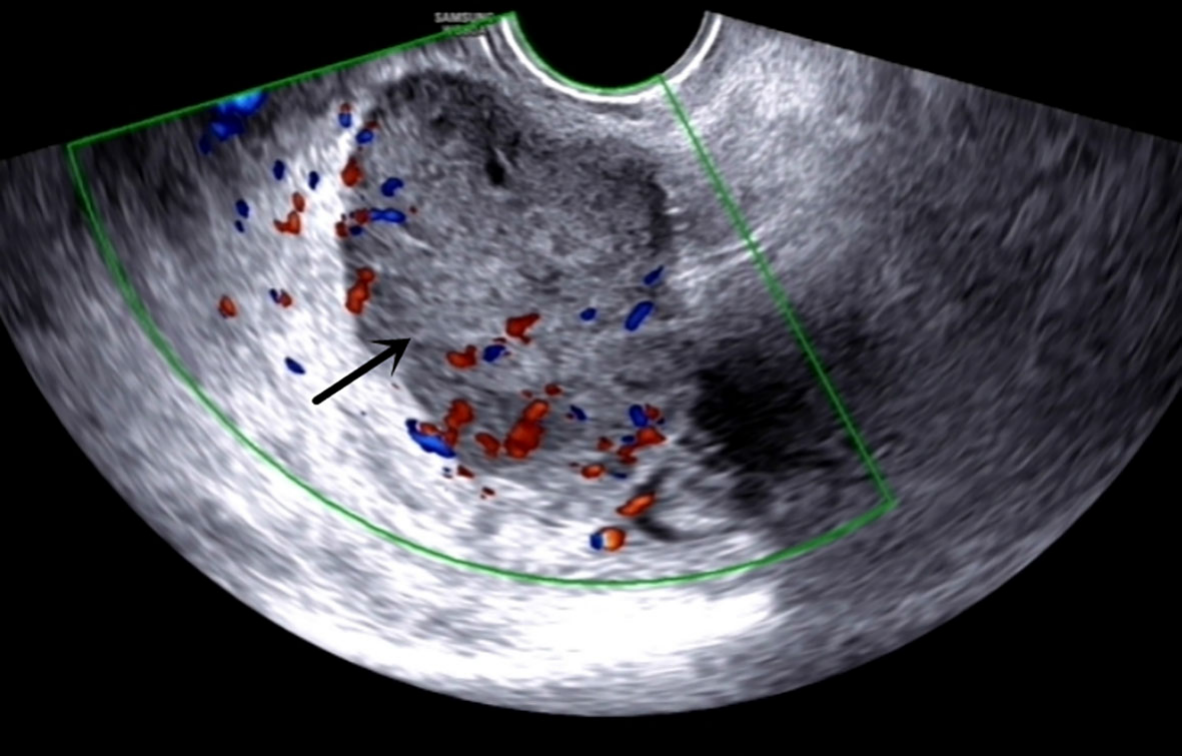
TVS: A 5.1 × 4.6 × 3.7 cm mass can be seen at the right rear of the cervical isthmus.

**Figure 2:**
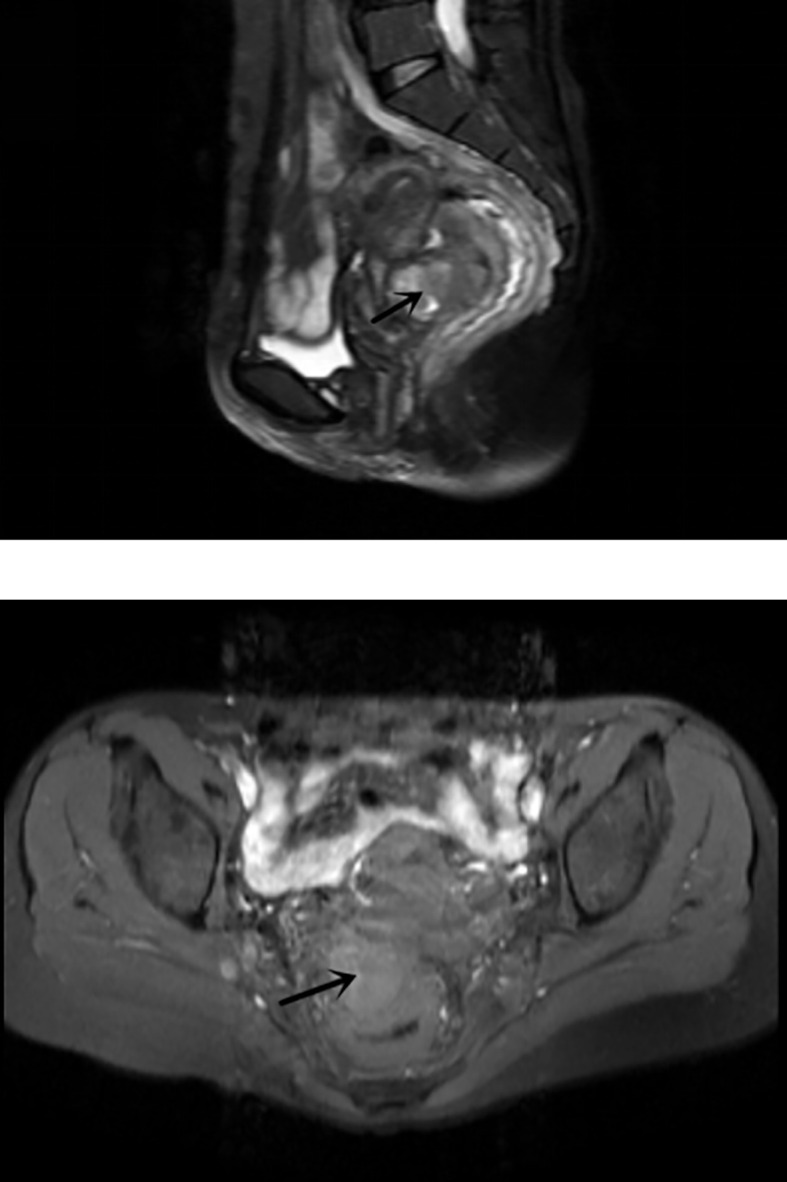
Pelvic MRI: Hybrid signal packet block at the right rear of the uterus.

**Figure 3:**
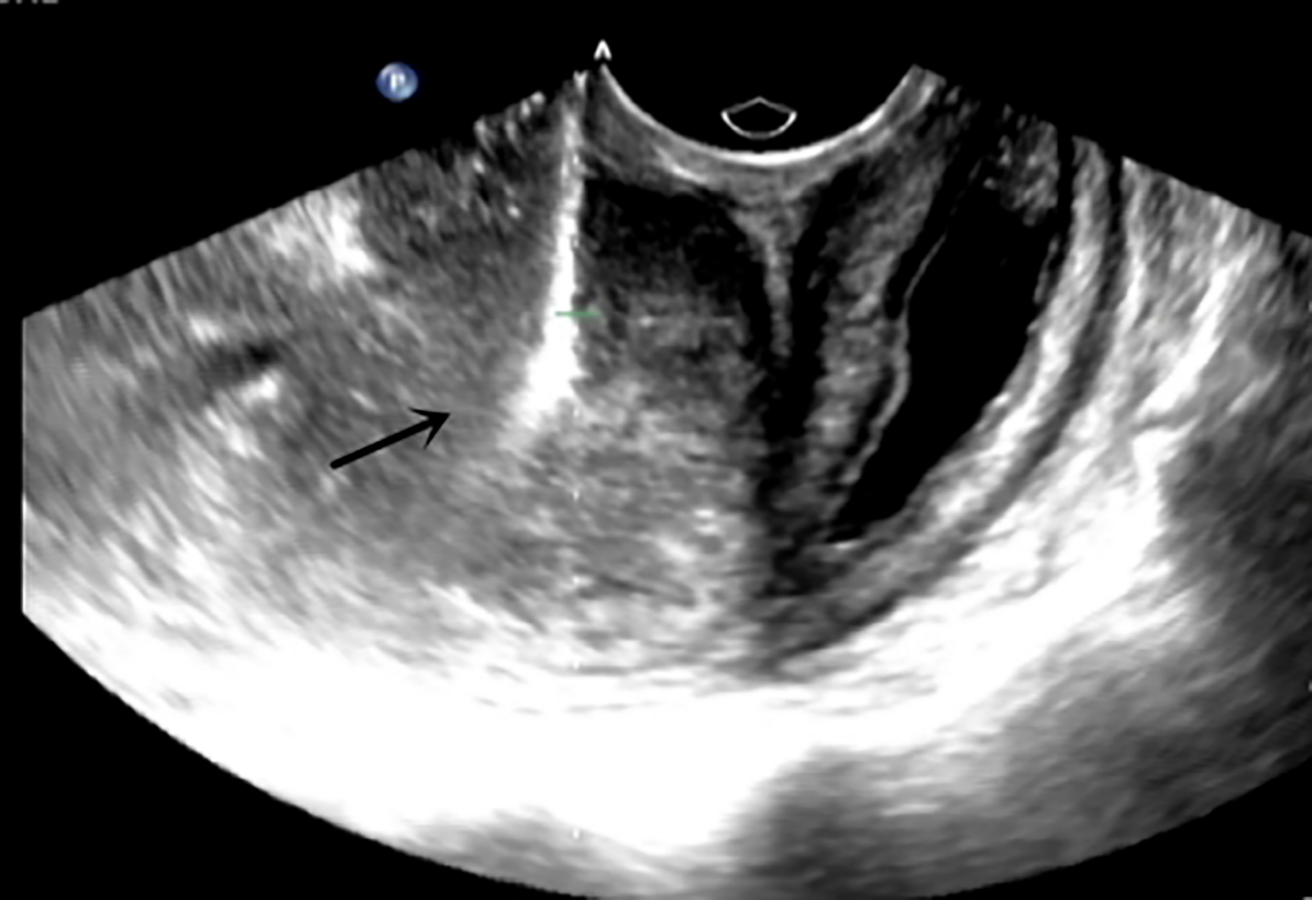
Ultrasound guided transvaginal pelvic mass biopsy.

**Figure 4:**
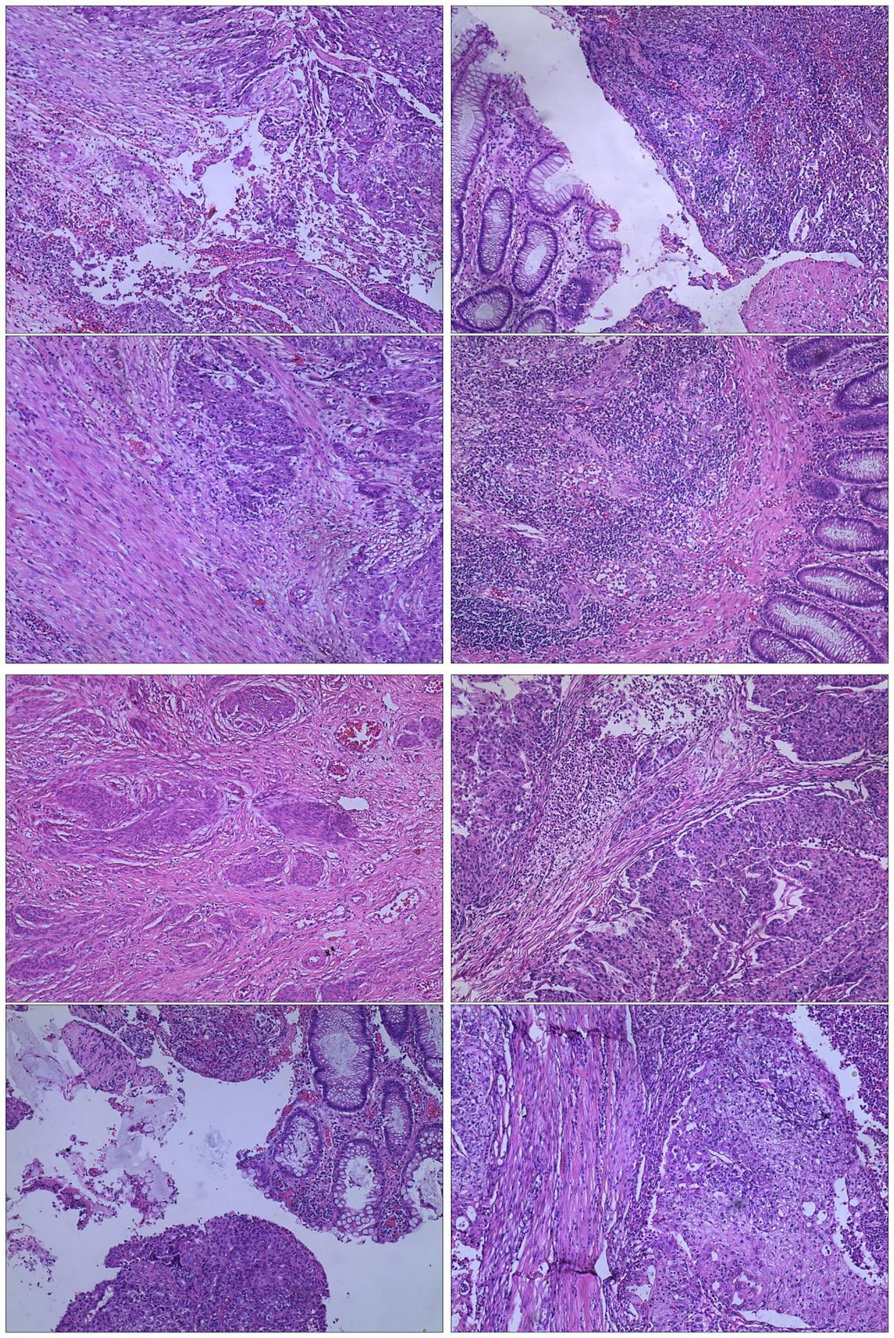
Histopathology of primary tumor.

**Figure 5:**
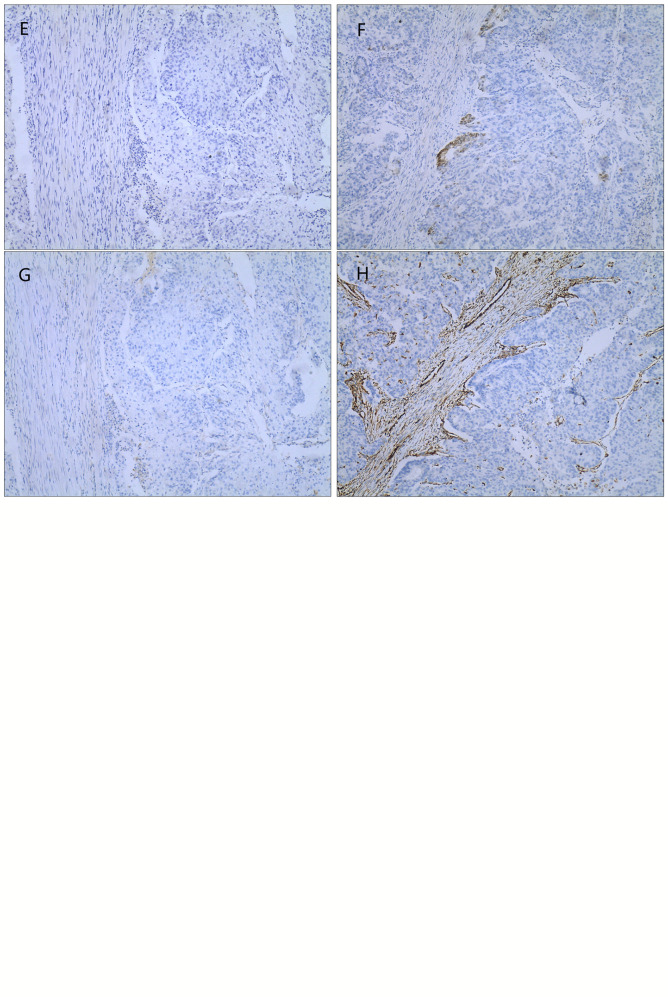
IHC(X10): **(A)** CDX-2(−); **(B)** ER(+); **(C)** CK7(+); **(D)** P16(+); **(E)** P40(−); **(F)** PR(+); **(G)** P63(−); **(H)** Vimentin(−).

The admission laboratory examination showed that the infection index was high and the patient was in a state of moderate anemia, CA125: 200.5 U/ml (normal <35.0). Gynecological examination indicated a tumor of about 5 cm in size with poor mobility which is between the uterine isthmus and rectum. TVS showed that a 5.1 × 4.6 × 3.7 cm mass was seen in the right posterior part of the uterine isthmus, with unclear boundary and mixed echo of cyst and blood flow signal could be detected by CDFI (**Figure 1**). A pelvic MRI indicated a mixed signal mass in the right posterior part of the uterus (**Figure 2**). After communicating with the ultrasound department and acquiring the informed consent of the patient, a pelvic tumor biopsy was performed under the guidance of an ultrasound through the vagina with local anesthesia (**Figure 3**). The pathology of the puncture biopsy revealed “adenocarcinoma, tending to endometrioid adenocarcinoma”. After full explanation with the patient, surgery was combined with transcystoscopic bilateral ureteral stent implantation, transabdominal rectal partial resection and anastomosis, radical uterectomy, left salpingo-oophorectomy, pelvic lymph node dissection, paraaortic lymph node sampling, omentectomy and appendectomy. In consideration of the special family history of the patient, LS was not excluded, and gene testing was carried out at the same time. The patients were given symptomatic treatment such as anti-inflammatory, fluid supplement, prevention of fungal infection and correction of anemia. More than ten days after operation, there was a little fecal tissue in the vaginal drainage tube. Since the digital rectal examination could touch the fistula, the anastomotic fistula was determined to be about 1 cm long, so the patient underwent temporary colostomy and rectal repair. Histopathological diagnosis suggested that “endometrioid adenocarcinoma (moderately differentiated) invaded the rectal wall and cervix, endometrium suggests focal complex hyperplasia, and there was no lymph node metastasis (**Figure 4**).” Immunohistochemistry staining (IHC) showed: CDX-2(−), ER(+), CK7(+), P16(+), P40(−), PR(+), P63(−), and Vimentin(−) (**Figure 5**). The results of gene detection indicated MSH2, MSH6 variation, PTEN, KRAS, NF1, KMT2C variation, and microsatellite are highly unstable.

The patients received 6 cycles of paclitaxel–carboplatin chemotherapy. During the treatment, grade II bone marrow suppression occurred, but was healed after symptomatic treatment. At follow-up date (10 months after surgery), CA125 has returned to normal level, and there is no obvious sign of tumor recurrence in imaging examination.

## Discussion and Conclusion

Endometriosis refers to the presence of endometrial tissue (glands and stroma) outside the uterine body, and is mostly a benign condition, commonly seen in women of childbearing age. Although endometriosis is a benign disease, it has malignancy-like behaviors such as invasion, implantation and recurrence (3). Endometriosis includes ovarian endometriosis, peritoneal endometriosis, DIE and endometriosis in other parts (such as abdominal wall incision, perineal incision and even invades the pleura). The overall malignant transformation rate of endometriosis is about 1.0%. Studies have shown that endometriosis is highly correlated with endometrioid carcinoma and clear cell carcinoma (1, 3), which mostly develop from ovarian endometriosis, and the malignant transformation of DIE is rare in clinic.

Endometriosis is defined as DIE when the lesion infiltrates ≥5 mm deep into the peritoneum or invades important organs such as intestine, ureter, and bladder. DIE malignant transformation is often misdiagnosed as rectal cancer or cervical cancer because it is located in the lowest position of pelvic cavity, which is in close proximity to rectum and cervical tissue (9, 10). DIE causes endometrial cells to flow back into the pelvic cavity. The combination of abnormal hormone levels, inflammatory factors, and immune mechanisms can lead to neovascularization. The lesions grow into deep peritoneal infiltrates, and stimulate the proliferation of deep fibrous connective tissue or make smooth muscle tissue form nodules in the uterus, ureter, vagina, rectum, and other parts. DIE can locally invade the surrounding structure, but rarely metastasize. Anglesio et al., researched DIE in terms of genome and confirmed that in 24 cases, 79% of DIE cases had somatic mutation events, and in all screened cases, 26% had statistically significant somatic mutations in known cancer driver genes (such as KRAS, PIK3CA, ARID1A) (11).

DIE is the most challenging disease in endometriosis, and sometimes medical therapy is enough to reduce symptoms and signs. Recent studies have shown that Dienogest can effectively treat endometriosis, reduce the size of endometriosis lesions and the postoperative recurrence rate of endometriosis (12). But in a large number of patients, a complete eradication, with nerve-sparing and vascular sparing approach is needed to restore the normal pelvic anatomy and its functions. The research of Raffaelli et al. showed that the application of Mesenteric vascular and nerve Sparing Surgery (MSS) in laparoscopic intestinal resection may be safe and effective, when partial intestinal resection is required. MSS can be combined with pelvic nerve sparing surgery as an effective method to improve intestinal symptoms (13).

As a special type of endometriosis, malignant transformation of DIE is relatively rare. We analyzed the reported cases of DIE malignant transformation in the recent 10 years, where the average age of onset is 48.75, and the age of the patient we reported is 49 years old, which is just about the average. We found that the major pathological type was endometrioid adenocarcinoma (87.5%). Only one of these cases recurred 11 years after being treated by chemotherapy, and pathological diagnosis of operation was clear cell carcinoma. Approximately 50% of the cases underwent adjuvant chemotherapy after surgical treatment, and there was no recurrence in the literature. The follow-up results of 75% of cases showed disease-free survival. Since DIE is in the deep infiltration position, the intestinal tract is often involved. When formulating the gynecological operation plan, surgery should be combined, and fistula should be performed if necessary, so as to reduce or avoid the occurrence of postoperative complications such as anastomotic fistula (**Table 1**).

**Table 1:** Summary of cases of DIE malignant transformation.

Reference	Age	Clinical presentation	Invasion site	Surgery	Pathological type	Adjuvant therapy	Outcome
Yang et al. (9)	57	vaginal bleeding, left lower abdominal pain	Douglas cul-de-sac	RAU + BAR + LE + OE + AE + PRR + OS	EC	CT	DFS: 12 mo
Marchand et al. (14)	63	pelvic discomfort, abdominal distension	rectovaginal septum	TR + UTER + BAR + LE + OE	EC		DFS:18 mo
Kim et al. (15)	40	No	rectovaginal septum	TR + RAU + PRR	EC	HRT	DFS: 8 mo
Ota et al. (16)	48	pelvic pain	Uterosacral ligament	UTER + OE + LE+	EC	CT	DFS: 2 yr
Mabrouk et al. (17)	36	abdominal discomfort	rectovaginal septum	RAU + LE + OE + AE + PRR + OS	EC	CT	DFS: 2 mo
Verma et al. (18)	49	sudden onset abdominal pain	Caecum	radical right hemicolectomy	EC	No	Loss of follow-up
Kondo et al. (19)	52	genital bleeding	vaginal	No	CCC	CT	R: 11 yr (Surgery: CCC)
Tarumi et al. (20)	45	frequent urination and miction pain	bladder	UTER partial bladder resection	EC	CT	DFS: 10 mo

RAU, Radical uterectomy; BAR, Bilateral adnexal resection; UAR, Unilateral adnexal resection; LE, Lymphadenectomy; UTER, Uterectomy; PRR, partial rectal resection; OS, ostomy; OE, Omentectomy; TR, Tumor resection; AE, Appendectomy; RT, Radiotherapy; CT, Chemotherapy; HRT, Hormone therapy; CRT, Chemo-and radio-therapy; DFS, Disease free survival; DOD, Dead of disease; LWD, Living with disease; R, Recurrence; EC, Endometrioid adenocarcinoma; CCC, clear-cell carcinoma.

At present, the pathogenesis of endometriosis-associated cancers is not fully understood. Inflammatory reaction and hormone imbalance may promote tumorigenesis. Reactive oxygen species (ROS) plays an important role in endometriosis by enriching free heme and catalytic iron, and may be one of the core pathogenesis of endometriosis-associated cancers (21). Specific miRNAs play an important role in tissue repair and transforming growth factors, cell growth, cell proliferation, apoptosis and angiogenesis and may be related to the malignant progression of endometriosis (22). Genetic background also plays an important role in the occurrence of endometriosis related cancer. The pattern of Loss Of Heterozygosity (LOH) in endometriosis associated carcinoma is similar to that in endometriosis, but the incidence of LOH is significantly higher than that in endometriosis (23).

Molecular mechanism of endometriosis-associated cancers has been confirmed to be related to gene mutations such as ARID1A, PI3K/AKT, β-catenin, PTEN and KRAS, etc. ARID1A mutation will result in the loss of function of tumor inhibition mechanism, and endometriosis is the first and only benign disease with missing ARID1A expression. ARID1A mutation is usually considered as an early event, but ARID1A inactivation by itself is not sufficient to lead to carcinogenic transformation of endometrial or ovarian surface epithelium. It is often a combination of mechanisms that trigger malignant transformation (24–27). PI3K plays an important role in cell growth, proliferation, movement, differentiation and angiogenesis (28). The activity of PI3K is regulated by oncogene PIK3CA and tumor suppressor gene PTEN. ARID1A mutations often coexist with mutations that lead to the activation of the PI3K/AKT signaling pathway, which is considered to be an early event of CCC (29–31). PTEN mutation has also been proved to be an important mechanism of endometriosis related cancer progression (31). It is also considered to be an early event in endometriosis related endometrioid adenocarcinoma and clear cell carcinoma. β-catenin mutation and over expression are very common in endometrioid adenocarcinoma. KRAS gene mutation is closely related to endometrioid adenocarcinoma and clear cell carcinoma, and is mainly involved in the advanced events of endometriosis related clear cell carcinoma (32).

The pathogenesis of endometriosis-associated cancers has also been linked to microsatellite instability, which may be related to LS. LS also known as a hereditary nonpolyposis colorectal cancer syndrome (HNPCC), is an autosomal dominant disease. Among female patients with LS, endometrial carcinoma and ovarian carcinoma are most closely associated, and can be regarded as the “sentinel” tumor of LS. In terms of histopathology, LS related endometrial carcinoma includes endometrioid adenocarcinoma and non endometrioid tissue types (clear cell carcinoma belongs to non endometrioid tissue type). Endometrioid adenocarcinoma and clear-cell carcinoma are also the main pathological types of endometriosis-associated cancers. In terms of pathogenesis, the pathogenesis of LS is a mismatch repair gene mutation, where the common mutant genes are MLH1, MSH2, MSH6, and PMS2. When mismatch repair genes are lost in function, duplicated coding gene fragment or noncoding gene fragment may mutate, resulting in microsatellite instability. This case was diagnosed as LS by gene detection for MSH2 mutation and other mutations in the solid cell germ line, combined with family history. At the same time, the patient we reported had a history of endometriosis and the pathology was endometrioid adenocarcinoma, which could suggest common mechanism pathways between LS and malignant transformation of endometriosis.

Kuo et al. found that among 48 cases of ovarian clear cell carcinoma, there were three cases also diagnosed as LS, suggesting that LS may be related to a certain frequency of ovarian clear cell carcinoma, and the mismatch repair gene mutation led to high-frequency microsatellite instability was the most closely related (1, 6). There have been studies examining the relationship between LS and endometrioid adenocarcinoma and clear cell carcinoma, but no systematic evaluation has been done, and the relationship between them may play a guiding role in the follow-up targeted therapy. The NCCN guidelines recommend that patients with EC should perform MMR/MSI status screening to determine the presence of LS. With the development of high-throughput sequencing technology, we have deepened our understanding on the pathogenesis of EC. Current research shows that adenomyosis may be associated to endometrial cancer (EC). There are several common molecular pathways of these two diseases (33, 34). In the future, we need more research to explore whether these tests can be applied to the malignant transformation of endometriosis and adenomyosis, even ovarian cancer. These tests may provide targeted treatment, immune checkpoint inhibitor treatment and other options for these malignant tumors.

Due to the malignant potential of endometriosis, special vigilance is needed in the process of diagnosis and treatment. TVS can initially determine the location, boundary and blood flow signal of tumor. A pelvic MRI can further show the origin of tumor and its relationship with surrounding tissues, and show a greater application prospect in the diagnosis of malignant transformation of endometriosis. It has certain clinical significance for judging benign and malignant, but it still has limitations. Ultrasound-guided tissue puncture can be used for pathological diagnosis, and the subsequent treatment can be decided according to the pathological type. CA125 can be used as a clinical reference index, but it is also generally increased in benign endometriosis (35, 36). When rectal invasion is suspected in DIE, diagnostic rectoscopy or colonoscopy should be performed, where an endoscopic biopsy may also be required for pathological examination. In this case, combined with the family historyof the patient, we should be on high alert for rectal cancer before operation. We can distinguish primary intestinal cancer and metastatic cancer according to IHC. IHC staining of CK7 and CK20 can distinguish endometrioid cancer and primary rectal cancer. Colorectal cancer is usually CK20 positive and CK7 negative, while gynecological tumors are usually CK20 negative and CK7 positive (9).

We should strengthen the management of patients with endometriosis with malignant potential. In the future, efforts should be made to develop effective and non-invasive screening tools to early identify women at risk of developing cancers. Malignant transformation of endometriosis should be fully evaluated before comprehensive surgical treatment. Currently, paclitaxel–carboplatin is the main chemotherapy. Targeted therapy and immunotherapy are available in advanced treatment through IHC and genetic testing.

In conclusion, malignant transformation of DIE invading surrounding tissues with LS is extremely rare, and we suggest that attention should be paid to the common molecular mechanism between malignant transformation of endometriosis and LS, so in the future the research between them may be carried out. In clinical treatment, we should strengthen the comprehensive management of malignant transformation of endometriosis, through evaluation of imaging and tumor marker, where surgery combined with chemotherapy seems to be the standard treatment. If necessary, immunohistochemistry and gene detection should be improved to provide follow-up targeted therapy and immunotherapy.

## Author Contributions

BXL and YangW are the common first authors, the major contributors in writing the manuscript. KRL edited the manuscript, and approved the final version. YueW and SML were responsible for reviewing the literature and collecting the information of the patients. All authors listed have made a substantial, direct, and intellectual contribution to the work and approved it for publication.

## Funding

This research received the support from a “Scientific research funding project of the Liaoning Provincial Department of Science and Technology (No.2020JH2/10300050)”.

## Conflict of Interest

The authors declare that the research was conducted in the absence of any commercial or financial relationships that could be construed as a potential conflict of interest.

## Publisher’s Note

All claims expressed in this article are solely those of the authors and do not necessarily represent those of their affiliated organizations, or those of the publisher, the editors and the reviewers. Any product that may be evaluated in this article, or claim that may be made by its manufacturer, is not guaranteed or endorsed by the publisher.
